# Investigating the effects of Liushen Capsules (LS) on the metabolome of seasonal influenza: A randomized clinical trial

**DOI:** 10.3389/fphar.2022.968182

**Published:** 2022-08-11

**Authors:** Qinhai Ma, Ruihan Chen, Jing Zeng, Biao Lei, Feng Ye, Qihua Wu, Zhengtu Li, Yangqing Zhan, Bin Liu, Bojun Chen, Zifeng Yang

**Affiliations:** ^1^ State Key Laboratory of Respiratory Disease, National Clinical Research Center for Respiratory Disease, Guangzhou Institute of Respiratory Health, The First Affiliated Hospital of Guangzhou Medical University, Guangzhou, Guangdong, China; ^2^ Faculty of Innovation Engineering, Macau University of Science and Technology, Taipa, Macao, China; ^3^ The Second Affiliated Hospital of Guangzhou University of Chinese Medicine, Guangzhou, Guangdong, China; ^4^ Guangzhou Laboratory, Guangdong, China; ^5^ State Key Laboratory of Quality Research in Chinese Medicine, Macau University of Science and Technology, Taipa, Macau, China

**Keywords:** influenza virus, liushen capsules, clinical trial, non-targeted metabolomics, Chinese traditional medicines

## Abstract

**Background:** Traditional Chinese Medicines (TCMs) are effective strategies for preventing influenza infection. Liushen Capsules can inhibit influenza virus proliferation, significantly mitigate virus-induced inflammation and improve acute lung injury *in vitro* or *in vivo*. However, the efficacy and safety of LS in clinical trials, and the role of LS in regulating metabolites in patients are not well known.

**Materials and methods:** A randomized, double-blind, placebo-controlled clinical trial was designed in this study. All participants were enrolled between December 2019 and November 2020. The efficacy and safety were assessed by primary efficacy endpoint ((area under the curve (AUC) analysis)) and secondary endpoint (individual scores for each symptom, remission of symptoms, and rates of inflammatory factors). The serum samples were collected from patients to detect the levels of inflammatory factors using RT-PCR and to identify metabolites using a non-targeted metabolomics ultra-performance liquid chromatography-tandem mass spectrometry (LC-MS).

**Results:** 81 participants from The Second Affiliated Hospital of Guangzhou University of Chinese Medicine and the First Affiliated Hospital of Guangzhou Medical University were completed the full study. After 14 days of intervention, the area under the curve (AUC) of the total symptom scores in LS group was significantly smaller than that in Placebo group (*p* < 0.001). Alleviation of sore throat, cough and nasal congestion in the LS group was significantly better than that in the Placebo group. The time and number to alleviation of symptoms or complete alleviation of symptoms in LS group was significantly better than that in Placebo group. The adverse effects of clinical therapy were slightly higher in LS group than in Placebo group, but there was no statistical difference. After 14 days of LS intervention, the levels of IL-1ra, Eotaxin, IFN-γ, IL-6, IL-10, IL-13, SCF and TRAIL in serum of participants with influenza infection were significantly decreased compared with Placebo group. It was observed that there were significant differences in the serum metabolic profiles between start- and end- LS groups. Further correlation analysis showed a potential regulatory crosstalk between glycerophospholipids, sphingolipids fatty acyls and excessive inflammation and clinical symptoms. Importantly, it may be closely related to phospholipid, fatty acid, arachidonic acid and amyl-tRNA synthesis pathway metabolic pathways.

**Conclusion:** The study showed there were no clinically significant adverse effects on LS, and a significant improvement in influenza-like symptomatology and inflammatory response in patients treated with LS. Further analysis showed that LS could significantly correct the metabolic disorders in the serum metabolite profile of the patients. This provided new insights into the potential mechanism of LS for the treatment of influenza.

## 1 Introduction

The influenza virus is the most common pathogen of respiratory viral infections and one of the one of the leading causes of viral pneumonia (Li et al., 2021). Influenza virus infection is a highly contagious respiratory disease resulting in high global morbidity and mortality, with a global disease burden estimated at 3 to 5 million cases per year. Globally, the level of influenza activity continued to rise, with positive influenza detections higher than in the same period in 2020 and lower than pre-COVID-19 pandemic levels. The influenza A (H3N2) subtypes and influenza B (Victoria) were the main ones (World Health Organization 2022). Although the global COVID-19 pandemic has altered or potentially reduced the annual circulation of influenza viruses worldwide, the impact of influenza viruses cannot be ignored. Influenza usually causes a simple respiratory infection, including fever, headache, muscle aches, chills, sweating, fatigue, sore throat, cough and nasal congestion that lasts for 3–14 days. Onset is usually rapid. A minority of patients, especially the elderly people, young children, and those with chronic lung disease, are at increased risk for complications from influenza. They may experience severe illness from viral or secondary bacterial pneumonia with extra-respiratory complications of the central nervous system or cardiovascular system (Peteranderl et al., 2016; Veldhuis Kroeze et al., 2021; Centers for Disease Control and Prevention, 2022). M2 ion channel inhibitor, Neuraminidase inhibitor, Endonuclease inhibitor and RNA-dependent RNA polymerase inhibitor are widely available for treatment of influenza virus. Despite the effectiveness of these antiviral agents in preventing influenza virus infection, the emergence of drug resistance restricts their application. Since 2013, influenza A virus subtypes circulating in the world have been significantly resistant to adamantanes (Dong et al., 2015). Currently, some circulating strains are resistant to Neuraminidase inhibitors such as peramivir and oseltamivir (Li et al., 2015). The newly emerged drug resistance to baloxavir and marboxi also remains a threat (Lampejo, 2020). Effective strategies for treatment of influenza virus are urgently needed.

Emerging respiratory infectious diseases are a huge challenge for scientists owing to lack of specific antiviral agents or vaccines. Traditional Chinese Medicine (TCMs) have been widely used in treatment of emerging respiratory infectious diseases in China such as influenza virus, SARS, SARS-CoV-2 and so on. Of note, TCMs are beneficial for the treatment of emerging respiratory infectious diseases such as Mexican Flu in 2009 and COVID-19 in China. They can effectively alleviate respiratory symptoms, reduce the time to symptom recovery, improve the prognosis of patients (Wang et al., 2011; Hu et al., 2021). Patient’s benefits from TCMs may be attributed to their antiviral, anti-inflammatory, antioxidant, and anti-apoptotic effects. TCMs were effective strategies for preventing influenza A virus (H1N1) pandemic in 2009 and they are beneficial for treating influenza virus infection. Since 2009, TCMs have been recommended for treatment of influenza infection in China (Ministry of Health China, 2009). But clinical trials should be done to fully figure out how well they work for treating the flu in a large group of people.

Liushen Capsules (LS), characterized by broad-spectrum antiviral and anti-inflammatory activity, have been recommended for treatment of influenza virus infection in China (Liu and Liu, 2022). Our previous studies found that LS could inhibit LPS-induced inflammation via NF-κB signaling pathway in RAW264.7 cells (Ma Qinhai, 2022). We also found that LS could inhibit SARS-CoV-2 proliferation and alleviate SARS-CoV-2 induced-inflammation *in vitro* and *in vivo* (Ma et al., 2022). Notably, our study found that LS could inhibit influenza A virus proliferation, significantly mitigate inflammation caused by influenza A virus via downregulation TLR4/NF-κB signaling pathway *in vitro* and *in vivo* and improve acute lung injury (ALI) caused by influenza A virus (Ma et al., 2020). In addition, LS could reduce inflammatory response and alleviate ALI caused by viral and secondary bacterial infection (Zhao et al., 2021). Therefore, LS is a promising agent for treatment of influenza infection. A randomized controlled clinical trial has been found that compared with Banlangen Granules, LS were superior in improving pharyngitis and reducing the use of acetyl aminophenol tablets in patients with influenza (Yong, 2021). To fully evaluate the safety and effectiveness of LS, however, double-blind, prospective, randomized controlled trials are needed.

Metabolomics provides a comprehensive analysis of lipids, organic acids, alkaloids and other metabolites within a given biological subject. By analyzing the differences between the drug-treated and placebo groups, the antiviral mode of action of drug can be elucidated, and potential antiviral targets for drug development can be identified (Gowda et al., 2008; Rabinowitz et al., 2011; Nuzzo et al., 2021).

The primary aim of the present study was to assess influenza improvement as measured by changes from baseline in clinical symptoms and changes in cytokines levels in LS after 14 days of treatment, and the safety of LS was assessed by the Common Terminology Criteria for Adverse Events (CTCAE). In addition, we further investigated the changes of plasma metabolites before and after LS intervention, and identified correlations between metabolites from patients and clinical symptoms or cytokines. Further clarified the possible anti-influenza virus mechanism of LS, and identified potential antiviral targets for LS.

## 2 Materials and methods

### 2.1 Study design

A randomized, double-blind, placebo-controlled clinical trial was designed in this study and was carried out at 2 sites (The Second Affiliated Hospital of Guangzhou University of Chinese Medicine and the First Affiliated Hospital of Guangzhou Medical University) in China. All participants were enrolled in this trial between December 2019 and November 2020. This study was approved by Ethics Committee of the First Affiliated Hospital of Guangzhou Medical University (Reference number: [2019] No. 56). The study was conducted under written informed consent from each participant in accordance with the provisions of the Declaration of Helsinki, Drug Administration Law, Drug Registration Regulation, Good Clinical Practice (GCP) and drug clinical trial regulations. This study was registered with the China Clinical Trial Registration Center (ChiCTR1900027401).

### 2.2 Inclusion and exclusion criteria

Participants were included in this study if they fulfilled any one of the following criteria: 1) female or male (18–65 years old); 2) with confirmed influenza infection (influenza A H1N1, influenza A H3N2 or influenza B virus) according to real-time polymerase chain reaction (PCR) or viral culture; 3) with acute onset (onset of any influenza symptom such as temperature ≥38.0°C) and duration of illness ≤48 h; 4) axillary temperature ≥38°C in the past 24 h; 5) the onset of one of the following systemic symptoms: headache, muscle aches, chills, sweating and fatigue; 6) the onset of one of the following respiratory symptoms: cough, sore throat and nasal congestion; 7) voluntary enrollment with written informed consent.

Participants who met any one of the following conditions were excluded from the study: 1) novel coronavirus infection with PCR-proven test; 2) Participants with bronchitis, pneumonia, pleural effusion and interstitial lesions, etc via chest imaging (radiograph or CT); 3) Abnormal leukocyte counts (>1×ULN or <1×LLN); 4) Coughing up purulent or bloody sputum or suffering from purulent tonsillitis; 5) Severe vomiting and diarrhea with signs of dehydration; 6) Rapid respiratory rate, dyspnea, cyanosis of lips and mouth; 7) Altered mental status (unresponsiveness, drowsiness, agitation, convulsions, etc; 8) BMI >30 kg/m^2^; 9) Patients with diabetes; severe underlying conditions: hematological disease, severe COPD (FEV1/EVC < (FEV1/EVC <70%, FEV1 <50% of expected value), bronchial asthma, bronchiectasis, respiratory failure, right heart failure; severe hepatic insufficiency (ALT or AST ≥3 ULN, total bilirubin ≥1.5 ULN); severe renal insufficiency (serum creatinine >177 μmol/L or 2 mg/dL); chronic congestive heart failure (cardiac function NYHA class III-IV); psychiatric patients; (10) Administration of drugs for treatment of influenza virus infection (oseltamivir, zanamivir, paramivir, famipiravir, abirater, amantadine, amantadine, etc.) within 7 days prior to enrollment; administration of traditional Chinese medicine or proprietary Chinese medicine with antiviral properties (Jinhua Qinggang capsules, Lianhua Qingwen capsules, Shufeng Jiedu capsules, Yin Qiao Jiedu capsules, etc); administration of LS capsules within 1 week prior to enrollment; 11) Allergy or hypersensitivity to the ingredients contained in the LS capsules; 12) Women who were breastfeeding and pregnant; Women who were planning a pregnancy within 6 months or had a positive urine pregnancy test; Men with fertility and sperm donation plans; 13) Immunodeficient patients (patients with malignancies, organ or bone marrow transplant recipients, AIDS patients and those taking immunosuppressive drugs within 3 months prior to the screening test); 14) Suspected or confirmed history of alcohol or drug abuse; 15) Patients who had participated in other drug clinical trials within 3 months prior to enrollment in this study; 16) Patients who had an acute respiratory infection, otitis media or sinusitis within 2 weeks prior to enrollment in this study; 17) Patients who had received seasonal influenza or novel influenza A H1N1 vaccine within 6 months prior to enrollment in this study; 18) Other patients who were deemed by the investigator to be inappropriate for this study.

### 2.3 Drug preparation

LS (Z20060207) was produced and provided by Suzhou Leiyunshang Medicine Pharmaceutical Co., Ltd. (Suzhou, China), was composed of 6 herbs including bezoar (the gallstone of bos taurus domesticus gmelin), cinobufagin toad venom (the excretion of venenum bufonis), pearl (the shell of pernulo), musk (the excretion of moschus), realgar, and borneol. Previous study indicated that LS contained 0.12% gamabufotalin, 0.10% arenobufagin, 0.26% telocinobufagin, 0.21% desacetylcinobufotalin, 0.25% bufotalin, 0.41% cinobufotalin, 0.27% bufalin, 0.70% resibufogenin, 0.68% cinobufagin, 1.81% cholic acid, 0.27% anserine deoxycholic acid, and 0.23% deoxycholic acid. The drug quality standards complied with the provisions of part I of the 2015 edition of the Chinese Pharmacopoeia (Li et al., 2019).

### 2.4 Interventions

Multicenter stratified block randomization method was employed to generate random numbers by SAS statistical analysis software (version 9.4). LS is a licensed drug, so drug administration is strictly based on the drug instructions. Participants were randomly treated with LS (1 capsule once) 3 times a day for 5 days versus LS simulants (1 capsule once) 3 times a day for 5 days. Data analysis was completed independently by professional statisticians.

### 2.5 Outcome measurements

The primary efficacy end point was assessed by an area under the curve (AUC) analysis of total symptom scores (fever, headache, muscle aches, chills, sweating, fatigue, sore throat, cough and nasal congestion). The participants were scored as no symptoms (0), mild symptoms 1), middle symptoms 2) and severe symptoms 3) according to the influenza symptom scoring criteria (Table 1) twice daily from enrolment (day 1) until day 6 or until day 14 if there were still symptoms present.

**TABLE 1:** Influenza symptom scoring criteria.

Symptoms	Scoring criteria			
	0(Absent)	1 (Mild)	2 (Moderate)	3 (Severe)
Fever	≤37.2°C	37.3 ∼ 37.9°C	38.0 ∼ 38.9°C	≥39°C
Headache	Absent	Mild pain, occasional onset	Moderate or persistent pain	Severe headache, cannot continue to work
Muscle soreness	Absent	Mild muscle soreness	Moderate	Severe muscle soreness, cannot continue to work and sleep
Chills	Absent	A slight sense of aversion to cold, do not need to add clothes	Aversion to cold, need to add clothes	Chills and shivers, need more clothes or more covers/bedding
Sweating	Absent	Slight sweating	Moderate	Excessive sweating
Fatigue	Absent	Lassitude	Grudgingly able to work	Severe fatigue, cannot continue to work
Sore throat	Absent	Mild sore throat	Pharyngeal dryness, pharyngalgia and odynophagia	Severe sore throat, dysphagia
Cough	Absent	Occasionally	Often	Frequent coughing during the day and night, cannot continue to work and sleep
Nasal congestion	Absent	Nasal incompetence on one side	Bilateral nasal incompetence	Bilateral nasal incompetence, open-mouth breathing, affecting sleep

Secondary endpoints were individual scores for each symptom, time to alleviation of symptoms and complete alleviation of symptoms (alleviation of symptoms defined as the time from the start of the trial regimen to the time when all influenza-related symptoms (Table 1) were rated by the patients as absent or mild; complete alleviation of symptoms defined as the time from the start of the trial regimen to the time when all influenza-related symptoms (Table 1) were rated by the patients as absent or mild for at least 24 h), rate of number of participants with remission of symptoms and complete remission of symptoms (remission of symptoms defined as when all influenza-related symptoms (Table 1) were rated by the patients as absent or mild; complete remission of symptoms defined as when all influenza-related symptoms (Table 1) were rated by the patients as absent or mild for at least 24 h), inflammatory factors.

### 2.6 Safety and adverse event monitoring

Adverse events were monitored from the time the subject signed the informed consent form and continued for up to 14 days following the subject’s last visit. The LS timing depended on the treatment tolerance measured by CTCAE-the standardized classification of adverse effects of clinical therapy. CTCAE used a range of grades from 1 to 5 (1-Mild, 2-Moderate, 3 -Severe, 4-Life-threatening, 5-Death).

### 2.7 Serum sample collection

The serum samples were tested for metabolites using a non-targeted metabolomics ultra-performance liquid chromatography-tandem mass spectrometry (LC-MS). The blood samples were kept at room temperature for 30 min for clotting. The clotted blood samples were centrifuged at 3000 g at 4°C for 20 min to obtain the supernatant serum and quickly stored at - 80°C for further processing.

### 2.8 Elisa assay

The Human IL-1ra, Human IL-6, Human IL-10, Human IL-13, Human Eotaxin, Human IFN-γ, Human SCF and Human TRAIL (BD Biosciences, San Jose, CA, United States) ELISA kits were used to measure plasma concentration of IL-1ra, IL-6, IL-10, IL-13, Eotaxin, IFN-γ, SCF and TRAIL, respectively. We used assay protocol and sample dilution in accordance with the manufacturers’ instructions.

### 2.9 Serum metabolome analysis using LC-MS

100 μl of the samples were added to the internal standard (L-2-chlorophenylalanine, 0.3 mg/ml, methanol) and vortexed for 10 s. And then 300 μl of protein precipitant was added to methanol-acetonitrile and vortexed for 1 min. The extract was sonicated in an ice-water bath for 10 min, and then left for 2 h at −20°C. The supernatant was separated by centrifugation for 10 min and stored at −80°C until LC-MS analysis.

A Dionex Ultimate 3000 RS UHPLC system fitted with Q-Exactive quadrupole-Orbitrap mass spectrometer (Thermo Fisher Scientific, Waltham, MA, United States) was used to analyze the metabolic profiling. An ACQUITY UPLC BEH C18 column (100 mm × 2.1 mm, 1.8 μm) was employed in both positive and negative modes. The binary gradient elution system consisted of (A) water (containing 0.1% formic acid, v/v) and (B) acetonitrile (containing 0.1% formic acid, v/v). The flow rate was 0.4 ml/min and the column temperature was 45°C. The mass range was from m/z 66.7 to 1,000.5 during the analysis. The resolution was set at 70,000 for the full MS scans and 35,000 for HCD MS/MS scans. The collision energy was set at 10, 20 and 40 eV. The QCs were injected at regular intervals (every 10 samples) throughout the analytical run to provide a set of data from which repeatability can be assessed.

### 2.10 Data processing and data analysis

The raw LC-MS data were analyzed using the progenesis QI software (Waters Corporation, Milford, United States), which was based on public databases such as HMDB (http://www.hmdb.ca/), LIPID MAPS (http://www.lipidmaps.org/) and self-built databases. The positive and negative data were combined to get a combined dataset which was imported into R ropes package. Principle component analysis (PCA) and (orthogonal) partial least-squares-discriminant analysis OPLS-DA were carried out to visualize the metabolic alterations among experimental groups. The differential metabolites were selected on the basis of the combination of a statistically significant threshold of variable influence on projection (VIP) values obtained from the OPLS-DA model and *p*-values from a two-tailed Student’s t test on the normalized peak areas, where metabolites with VIP values larger than 1.0 and *p*-values less than 0.05 were considered as differential metabolites. The correlation between metabolites and clinical symptoms, metabolites and inflammatory factors was determined using Pearson correlation analysis (PCC). As well, the analysis of potential biomarkers and metabolic pathways was carried out by HMDB (http://www.hmdb.ca/) and KEGG (http://www.kegg.jp/kegg/pathway.html) databases.

### 2.11 Statistical analysis

All the data were presented as mean ± SD. The Mann-Whitney U test or Independent-samples T test was performed to compare the LS group with the Placebo group. Cytoscape (version 3.3.0) was used to construct PCC network. Categorical data were analyzed with Fisher’s exact test. *p*-values < 0.05 was considered to be statistically significant. SAS 9.2 software was used for the statistical analysis.

## 3 Results

### 3.1 Patient recruitment and baseline characteristics

**FIGURE 1:**
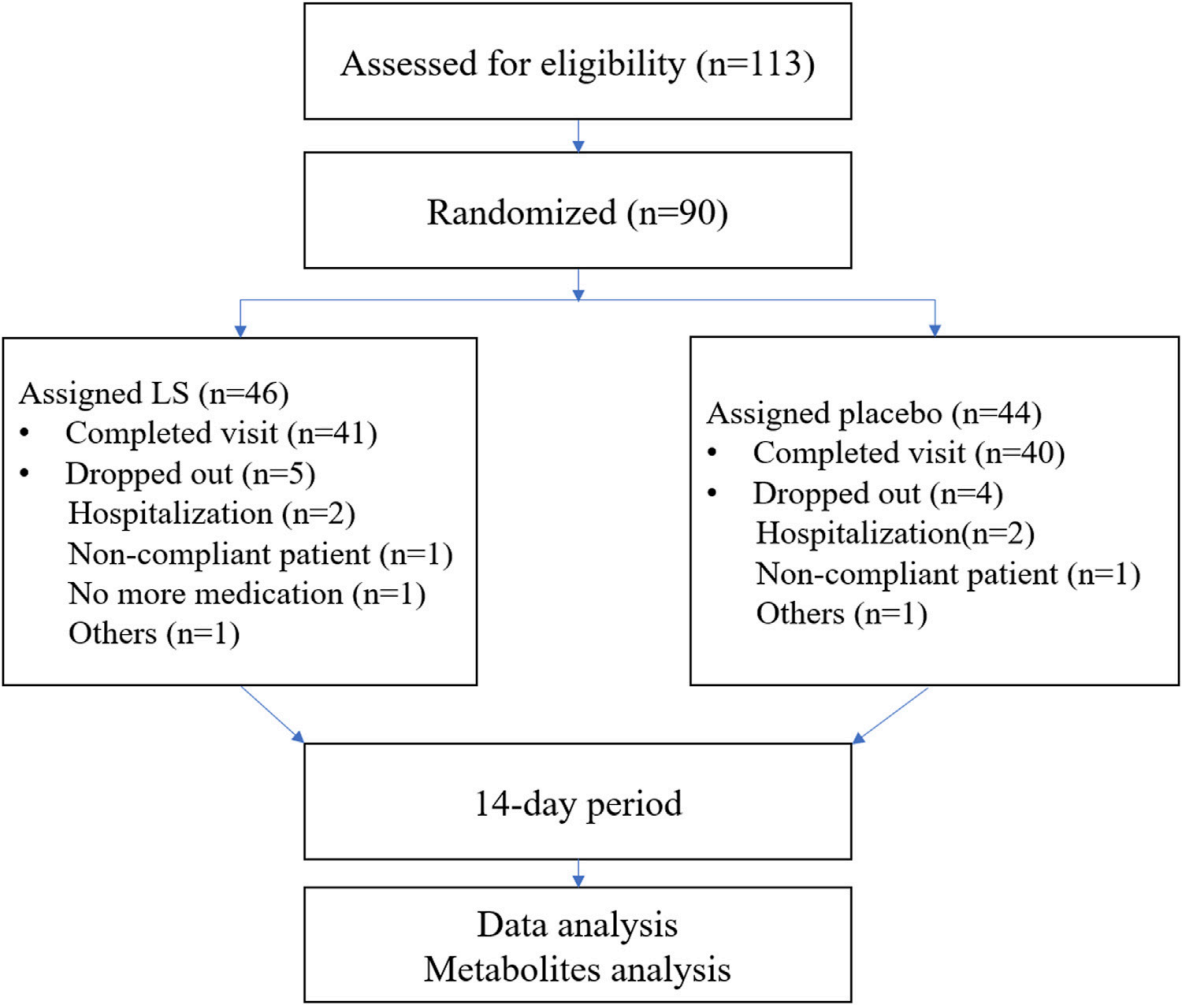
Schematic view of participant flow.

A total of 113 potential participants were screened from the First Affiliated Hospital of Guangzhou Medical University and The Second Affiliated Hospital of Guangzhou University of Chinese Medicine. 90 participants were eligible for the trial. The subjects were randomized into the two groups including LS group and Placebo group. 81 participants completed the full study, while 9 subjects dropped out (Figure 1). All the data were analyzed using Full Analysis Set (FAS). No relevant differences in demographic or clinical characteristics were noted between those assigned to LS group and those assigned to Placebo group (Table 2).

**TABLE 2:** General information on participants in the two groups.

Items	LS (*N* = 46)	Placebo (*N* = 44)	*p* value
Age (year, Mean ± Std)	21.50 ± 3.75	21.20 ± 4.42	0.7328
Sex (Man	28 (60.87%)	21 (47.73%)	0.2899
Female)	18 (39.13%)	23 (52.27%)	
Height (cm, Mean ± Std)	168.45 ± 7.50	166.33 ± 7.92	0.1963
Weight (kg, Mean ± Std)	59.92 ± 11.27	55.10 ± 8.75	0.0263
BMI (kg/m^2^, Mean ± Std)	21.00 ± 3.08	19.81 ± 2.02	0.0335
Body temperature (°C, Mean ± Std)	38.48 ± 0.68	38.61 ± 0.78	0.4322
Current smoker (No.%, Mean ± Std)	6 (13.04%)	1 (2.27%)	0.1109
Duration of influenza illness (h, Mean ± Std)	22.65 ± 12.18	26.55 ± 9.57	0.1005
Highest body temperature within 24 h before enrollment (°C, Mean ± Std)	38.74 ± 0.52	38.92 ± 0.53	0.1099
Influenza vaccination history	46 (100.00%)	44 (100.00%)	—
Relevant treatment history	46 (100.00%)	44 (100.00%)	—
Respiratory diseases	2 (4.35%)	0 (0.00%)	—
Other systemic diseases	1 (2.17%)	0 (0.00%)	—
Rapid virus antigen detection	Positive (Flu A)		0.3554
	45 (97.83%)	41 (93.18%)	
	Positive (Flu B)		
	1 (2.17%)	3 (6.82%)	

### 3.2 Primary outcome

After 14 days of intervention, the area under the curve (AUC) of the total influenza symptom scores in LS group was significantly smaller than that in Placebo group (*p* < 0.001). The result showed that the LS group could relieve participant’ influenza symptoms faster (Table 3).

**TABLE 3:** The AUC of total influenza symptom scores in two groups of participants (FAS).

		LS (N = 46)	Placebo (N = 44)	Statistic	*p* value
AUC	N(Nmiss)	45 (1)	41 (3)	t = 4.32	<0.001
	Mean ± Std	245.40 ± 174.08	416.78 ± 193.65	—	—
	95%CI	(193.10,297.70)	(355.66,477.91)	—	—
	Min ∼ Max	26.5–862.5	72.5–1,057.5	—	—

### 3.3 Secondary outcomes

Alleviation of sore throat and nasal congestion on visit 1 in the LS group was significantly lower than that in the Placebo group (−0.98 ± 0.87 vs. −0.44 ± 1.00, *p* = 0.0090; −0.47 ± 0.84 vs. 0.02 ± 1.15, *p* = 0.0256), respectively. Alleviation of cough on visit 1 and visit 2 in the LS group was significantly lower than that in the Placebo group (−0.42 ± 0.72 vs. 0.15 ± 0.76, *p* = 0.0006; −0.81 ± 0.80 vs. −0.23 ± 0.81, *p* = 0.0018), respectively (Table 4).

**TABLE 4:** Change values of sore throat, cough and nasal congestion symptoms from baseline between LS and Placebo groups (FAS).

	Items	LS (N = 46)	Placebo (N = 44)	Statistic	*p* value
Sore throat visit 1	N(Nmiss)	45 (1)	41 (3)	t = 2.68	0.0090
	Mean ± Std	−0.98 ± 0.87	−0.44 ± 1.00	—	—
	95%CI	(−1.24, −0.72)	(−0.76, −0.12)	—	—
	Min ∼ Max	−3∼1	−3∼3	—	—
Sore throat visit 2	N(Nmiss)	42 (4)	39 (5)	t = 0.59	0.5557
	Mean ± Std	−1.07 ± 0.92	−0.95 ± 0.94	—	—
	95%CI	(-1.36, -0.78)	(-1.25, -0.64)	—	—
	Min ∼ Max	−3∼1	−3∼1	—	—
Cough visit 1	N(Nmiss)	45 (1)	41 (3)	t = 3.55	0.0006
	Mean ± Std	-0.42 ± 0.72	0.15 ± 0.76	—	—
	95%CI	(−0.64, -0.21)	(−0.09,0.39)	—	—
	Min ∼ Max	−2∼1	−1∼2	—	—
Cough visit 2	N(Nmiss)	42 (4)	39 (5)	t = 3.23	0.0018
	Mean ± Std	−0.81 ± 0.80	−0.23 ± 0.81	—	—
	95%CI	(−1.06, -0.56)	(−0.49,0.03)	—	—
	Min ∼ Max	−3∼0	−1∼2	—	—
nasal congestion visit 1	N(Nmiss)	45 (1)	41 (3)	t = 2.27	0.0256
	Mean ± Std	−0.47 ± 0.84	0.02 ± 1.15	—	—
	95%CI	(−0.72, -0.21)	(−0.34,0.39)	—	—
	Min ∼ Max	−2∼1	−2∼3	—	—
nasal congestion visit 2	N(Nmiss)	42 (4)	39 (5)	t = 1.14	0.2597
	Mean ± Std	−0.71 ± 0.74	−0.51 ± 0.85	—	—
	95%CI	(−0.95, −0.48)	(−0.79, −0.24)	—	—
	Min ∼ Max	−3∼0	−3∼1	—	—

The time to alleviation of symptoms in LS group (30.00 ± 16.98 h) was significantly shorter than that in the Placebo group (40.51 ± 25.77 h) (*p* = 0.0296). The time to complete alleviation of symptoms in LS group (30.07 ± 17.41 h) was significantly shorter than that in the Placebo group (39.90 ± 21.91 h) (*p* = 0.0392) (Table 5).

**TABLE 5:** The remission time and complete remission time of symptoms in LS and Placebo group (FAS).

	Items	LS (N = 46	Placebo (N = 44)	Statistic	*p* value
time to alleviation of symptoms	N(Nmiss)	45 (1)	37 (7)	t = 2.22	0.0296
	Mean ± Std	30.00 ± 16.98	40.51 ± 25.77	—	—
	95%CI	(24.90,35.10)	(31.92,49.10)	—	—
	Min ∼ Max	9–97	11–114	—	—
time to complete alleviation of symptoms	N(Nmiss)	42 (4)	29 (15)	t = 2.10	0.0392
	Mean ± Std	30.07 ± 17.41	39.90 ± 21.91	—	—
	95%CI	(24.65,35.50)	(31.56,48.23)	—	—
	Min ∼ Max	9–97	11–87	—	—

The rate of number of participants with complete remission of symptoms on visit 1 and visit 2 in LS group (50.00 vs. 22.73%, *p* = 0.0090) was significantly higher than that in the Placebo group (91.30 vs. 65.91%, *p* = 0.0041) (Table 6).

**TABLE 6:** The rate of number of participants with complete remission of symptoms in LS and Placebo group (FAS).

		LS (N = 46)	Placebo (N = 44)	Statistic	*p* value
Visit 1	complete alleviating symptoms	23 (50.00)	10 (22.73)	Exact probability	0.0090
	alleviating symptoms, not complete	23 (50.00)	34 (77.27)	—	—
	total	46 (100.00)	44 (100.00)	—	—
Visit 2	complete alleviating symptoms	42 (91.30)	29 (65.91)	Exact probability	0.0041
	alleviating symptoms, not complete	4 (8.70)	15 (34.09)	—	—
	total	46 (100.00)	44 (100.00)	—	—

### 3.4 Safety and adverse events

Adverse events were reported in 71.11% of LS group and 59.09% of Placebo group and no significant difference was observed between the two groups (*p* = 0.234) (Table 7). But neither was considered to be related to the trial regimen by investigators who were unaware of the trial group assignments. There were no serious adverse events and no adverse events that were associated with cessation of the trial regimen.

**TABLE 7:** Adverse events in LS and Placebo group during trial.

Event	LS (N = 46)	Placebo (N = 44)
Any adverse event	32 (71.11)	26 (59.09)
Vomiting	17 (53.13)	2 (7.69)
Nausea	16 (50.00)	5 (19.23)
Cough and sputum	11 (34.38)	13 (50.00)
Diarrhea	7 (21.88)	7 (26.92)
Stomach discomfort	3 (9.38)	2 (7.69)
Nasal congestion	1 (3.13)	—
Gastrointestinal discomfort	1 (3.13)	1 (3.85)
Pneumonia	1 (3.13)	1 (3.85)
Abdominal rash	1 (3.13)	—
bloating	1 (3.13)	1 (3.85)
Mouth ulcers	1 (3.13)	—
Urine occult blood 3+	1 (3.13)	—
Fatigue	1 (3.13)	1 (3.85)
Subconjunctival hemorrhage	1 (3.13)	—
Upper respiratory tract infection	1 (3.13)	1 (3.85)
Dizziness	1 (3.13)	—
Bacterial Infections	1 (3.13)	—
Sore Throat	1 (3.13)	1 (3.85)
Foreign body sensation in the left eye	1 (3.13)	—
Decrease in white blood cells	—	1 (3.85)
Urinary Infections	—	1 (3.85)
Elevated urine leukocytes	—	1 (3.85)
Stabbing pain in the chest	—	1 (3.85)

### 3.5 LS reduced the level of inflammatory factors in participants

**FIGURE 2:**
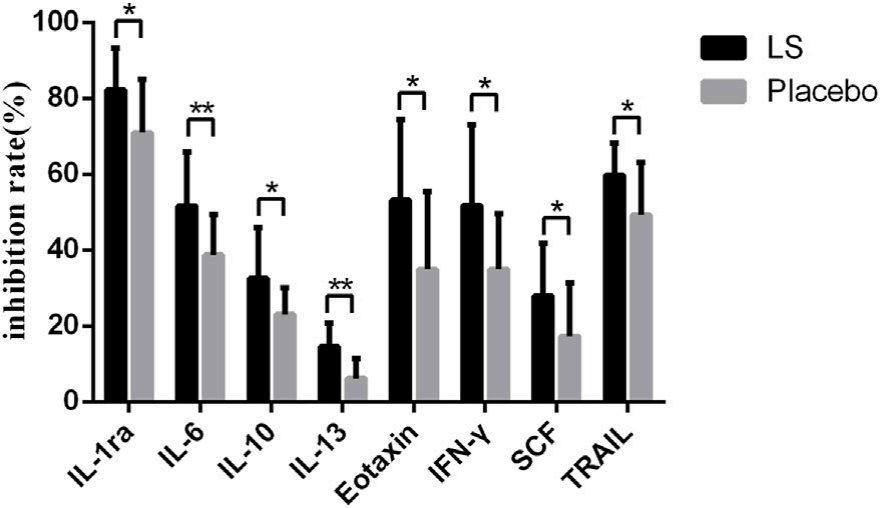
Inflammatory factors in the start- and end-LS groups.

After 14 days of LS intervention, the levels of IL-1ra, IL-6, IL-10, IL-13, Eotaxin, IFN-γ, SCF and TRAIL in serum of participants with influenza infection were significantly decreased compared with Placebo group (*p* < 0.05 or *p* < 0.01) (Figure 2).

### 3.6 LS modulated the metabolome in plasma

**FIGURE 3:**
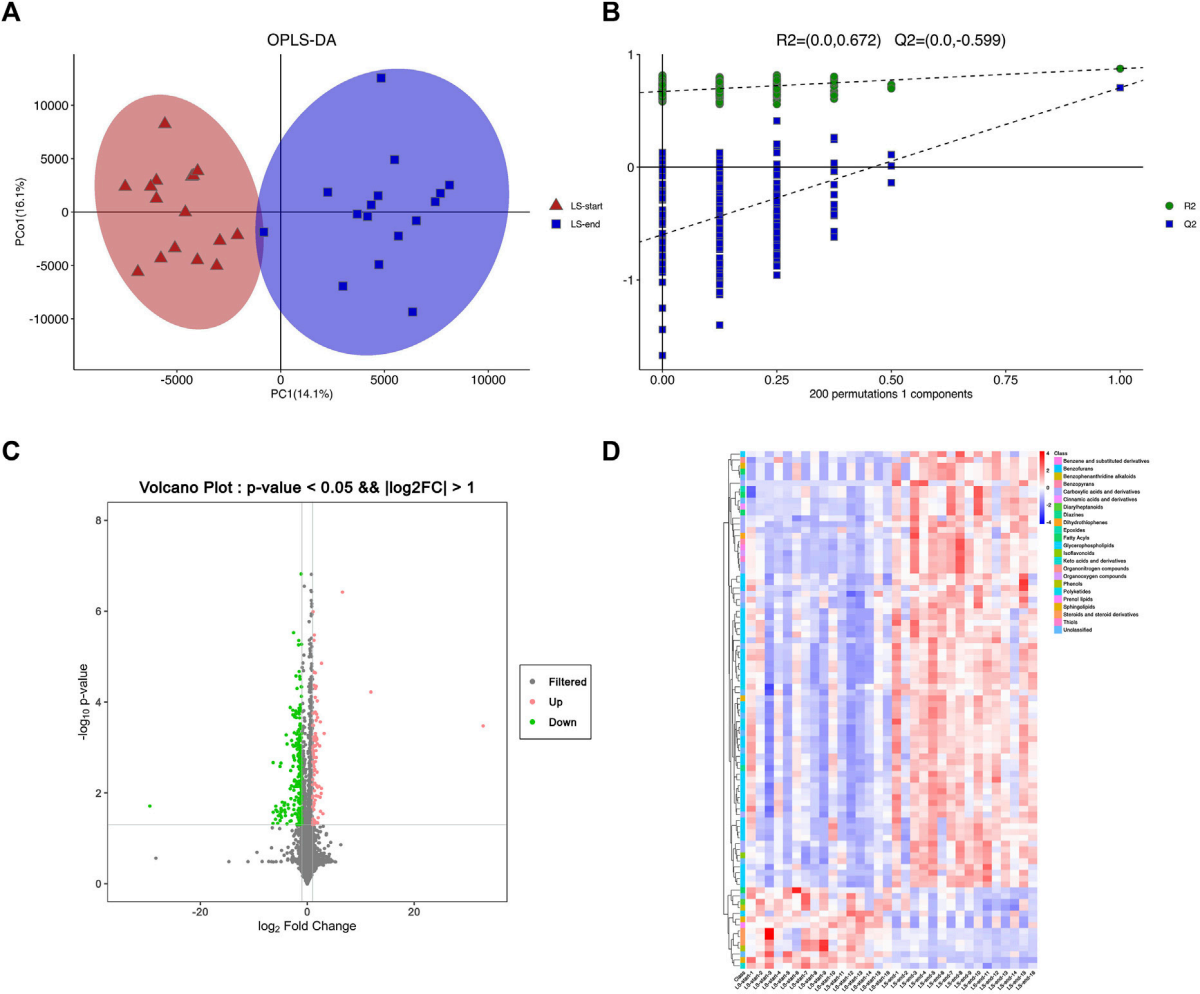
Serum metabolomics was used to quantify metabolites in the start- and end- LS groups. **(A)** OPLS-DA plot showing the spatial division between start- and end- LS groups. **(B)** Alignment diagram of correlation coefficients of OPLS-DA model. **(C)** Volcano plot showing the metabolites that differed cumulatively and significantly changed in start- and end- LS groups. **(D)** Heat map of the association between 89 metabolites and start/end LS intervention.

The two groups of serum samples were further analyzed using non-targeted LC-MS metabolomics approach. We successfully quantified 11,630 metabolites in positive or negative ion mode (Supplement Table S1). The ions with variable projection importance (VIP) values ​​> 1.0 were considered as potential differentially expressed metabolites. We successfully screened 202 metabolites with (VIP) score >1, which were significantly different between LS-start and LS-end groups (*p* < 0.05) (Supplement Table S2). The OPLS-DA score plot showed significant discriminatory ability between the two groups with R2X = 0.42, R2Y = 0.874, Q2Y = 0.705, indicating the predictive and reliable nature of the model (Figure 3A). The two OPLS-DA model correlation coefficients were used to validate the array plots of the model (Figure 3B). The volcano and S-plot analysis revealed a significant difference between LS-start and LS-end groups, 136 were up-regulated and 66 were down-regulated (VIP>1, *p* < 0.05) (Figure 3C). A total of 89 metabolites with VIP >1, *p* < 0.001 were listed in Supplement Table S3, plotted as a heatmap (Figure 3D). Glycerophospholipids, including PIM1 (18:0/16:2 (9Z,12Z)), PIM1 (17:0/18:0), PA (8:0/13:0) and lysope (18:2 (9Z,12Z)/0:0); carboxylic acids and derivatives, including glutaminyllysine and glutaminylaspartic acid; sphingolipids, including SM(d16:1/16:0) and GM4 (d18:1/16:0); fatty acyls, including avocadene 1-acetate showed higher abundance in the LS-end group than that in the LS-start group. Sphingolipids, including sphinganine 1-phosphate and SM(d16:1/18:0); glycerophospholipids, including PC (14:0/20:3 (5Z,8Z,11Z)); fatty acyls, including 3,4-dimethyl-5-carboxyethyl-2-furanpentanoic acid in the LS-end group, were less abundant than in the LS-start group.

### 3.7 LS modulated the metabolic pathways

**FIGURE 4:**
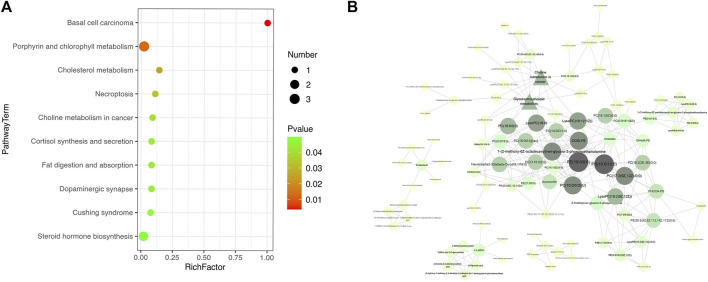
Pathway analysis was used to enrich the metabolic pathways of differential metabolites start- and end- LS treatment. **(A)** KEGG pathway showing the differential metabolic pathways in start- and end- LS groups. **(B)** Correlation analysis of serum metabolites with significantly difference. Only metabolite correlations with |PCC| ≥ 0.9 was considered. Circle indicated metabolites and triangles indicated metabolic pathways, with darker colors indicating higher correlations.

According to the KEGG database, as shown in Figure 4A, 19 metabolic pathways were disturbed before and after LS treatment (*p* < 0.05), including choline metabolism and central carbon metabolism in cancer, caffeine metabolism, D-arginine and D-ornithine metabolism, glycerophospholipid metabolism, sphingolipid metabolism, ether lipid metabolism, arginine and proline metabolism and mTOR signaling pathway, etc.

Further metabolomic pathway analysis revealed 89 metabolites and 11 metabolic pathways in the presence of the most obvious metabolic disturbance (|PCC|>0.9). Therefore, caffeine metabolism, D-arginine and D-ornithine metabolism, valine, leucine and isoleucine biosynthesis, arginine and proline metabolism and mTOR signaling pathway were filtered out. Meantime, ether lipid metabolism, sphingolipid metabolism, glycerophospholipid metabolism, cortisol synthesis and secretion, steroid hormone biosynthesis, choline metabolism and aminoacyl-tRNA biosynthesis may be the key pathways of LS to treat influenza. The main metabolites included glycerophosphocholines, glycerophosphoethanolamines, glycerophosphoserines, oxidized glycerophospholipids, flavonoids (Figure 4B).

### 3.8 Correlation analysis of clinical symptoms and metabolism

Influenza usually causes a range of symptoms, including fever, headache, muscle aches, chills, sweating, fatigue, sore throat, cough and nasal congestion. We obtained correlations between clinical symptoms and differential metabolites using cytoscape mapping (|PCC|> 0.4, *p* < 0.001). Pathway analysis revealed that the metabolic pathways were highly correlated with clinical symptoms (eg., fever, headache, muscle aches, chills, sweating, fatigue, sore throat and cough). Eight clinical symptoms were closely associated with metabolites involved in choline metabolism in cancer, arginine biosynthesis, D-arginine and D-ornithine metabolism, glycerophospholipid metabolism, valine, leucine and isoleucine biosynthesis, sphingolipid metabolism, ether lipid metabolism, central carbon metabolism in cancer, arginine and proline metabolism and mTOR signaling pathway. Mainly enriched in lipids and lipid-like molecules and organic acids and derivatives. Specifically, in the LS group, 77 correlations were observed between fever and metabolites; 78 correlations were observed between headache and metabolites; 70 correlations were observed between sore throat and metabolites; 77 correlations were observed between chills and metabolites; 54 correlations were observed between fatigue and metabolites; 41 correlations were observed between muscle ache and metabolites; 11 correlations were observed between cough and metabolites; 4 correlations were observed between sweating and metabolites (Supplement Table S4).

**FIGURE 5:**
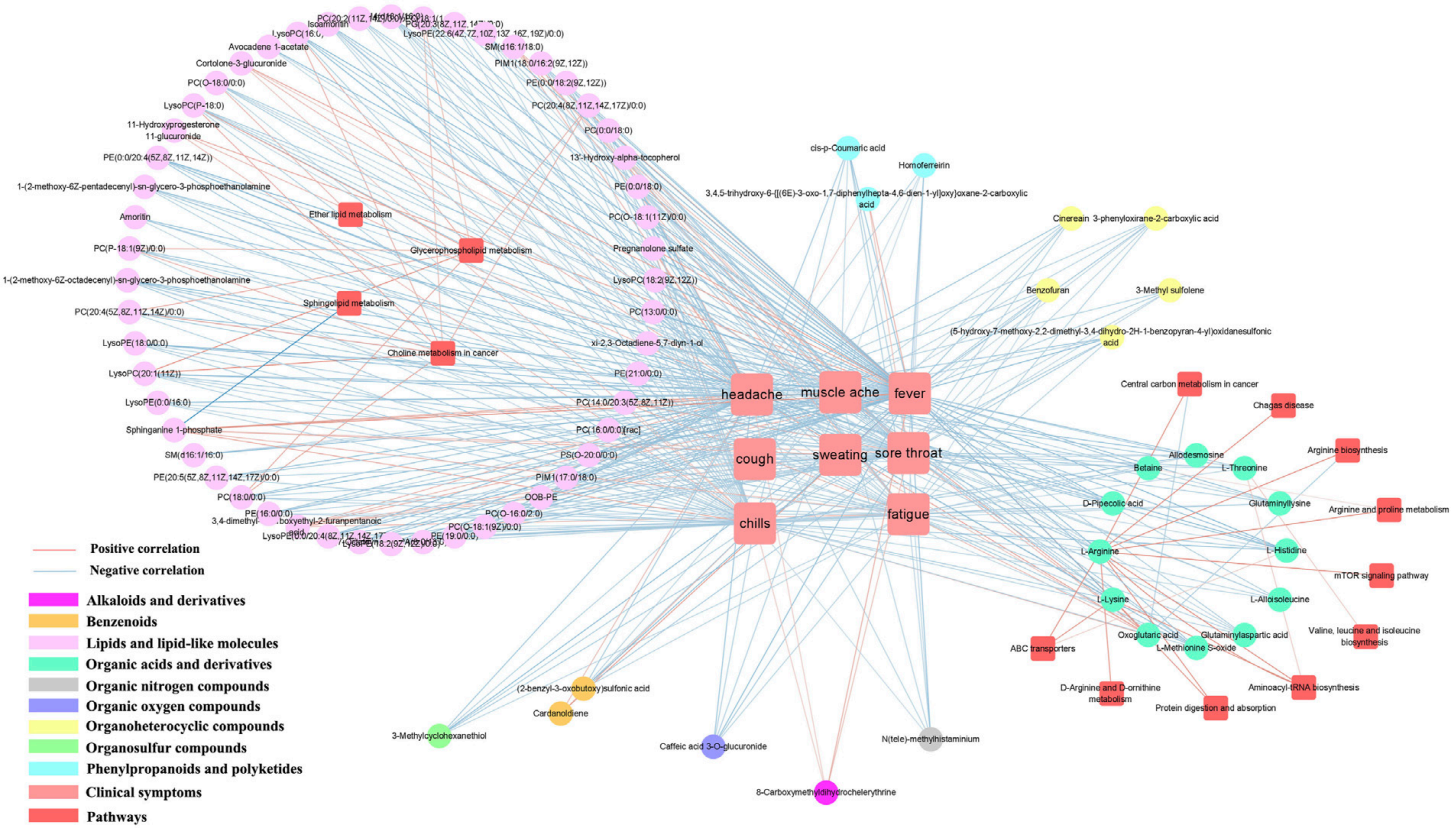
Metabolite-clinical symptom correlation in serum samples. Only metabolite correlations with |PCC| ≥ 0.9 and *p* < 0.001 were considered.

The majority of metabolites were negatively correlated with clinical symptoms, with the exception of SM(d16:1/18:0) and sphinganine 1-phosphate in phospholipid metabolites; cortolone-3-glucuronide and 11-hydroxyprogesterone 11-glucuronide in steroidal glycoside metabolites; pregnanolone sulfate; fatty acids and conjugates (3,4-dimethyl-5-carboxyethyl-2-furanpentanoic acid); glycerophosphocholines (PC(14:0/20:3 (5Z,8Z, 11Z))); phenylpropanoids and polyketides (3,4,5-trihydroxy-6-{[(6E)-3-oxo-1,7-diphenylhepta-4,6-dien-1-yl]oxy}oxane-2 -carboxylic acid); alkaloids and derivatives (8-carboxymethyldihydrochelerythrine); benzenoids (cardanoldiene); gamma-keto acids and derivatives (oxoglutaric acid) were positively correlated. However, PC (14:0/20:3 (5Z,8Z, 11Z)) and 13′-Hydroxy-alpha-tocopherol were negatively correlated with sweating (Figure 5).

### 3.9 Correlation analysis of cytokines and metabolism

Influenza usually causes a range of inflammatory, and we obtained correlations between inflammatory factors and differential metabolites using cytoscape mapping (|PCC|> 0.4, *p* < 0.001). Pathway analysis revealed that the metabolic pathways were highly correlated with inflammatory factors (eg., TRAIL, IL-6, IL-1ra, IFN-γ, IL-13, IL-7, SCF). The metabolic pathways involved in the 7 inflammatory factors were the same as the metabolic pathway network of clinical symptoms-metabolites. They also mainly enriched in lipids and lipid-like molecules and organic acids and derivatives. Specifically, in the LS group, 72 correlations were observed between TRAIL and metabolites, 72 correlations were observed between IL-6 and metabolites, 69 correlations were observed between IL-1ra and metabolites, 51 correlations were observed between IFN-γ and metabolites, 35 correlations were observed between IL-13 and metabolites, 20 correlations were observed between IL-7 and metabolites, 15 correlations were observed between SCF and metabolites, 11 correlations were observed between eotaxin and metabolites (Supplement Table S5).

**FIGURE 6:**
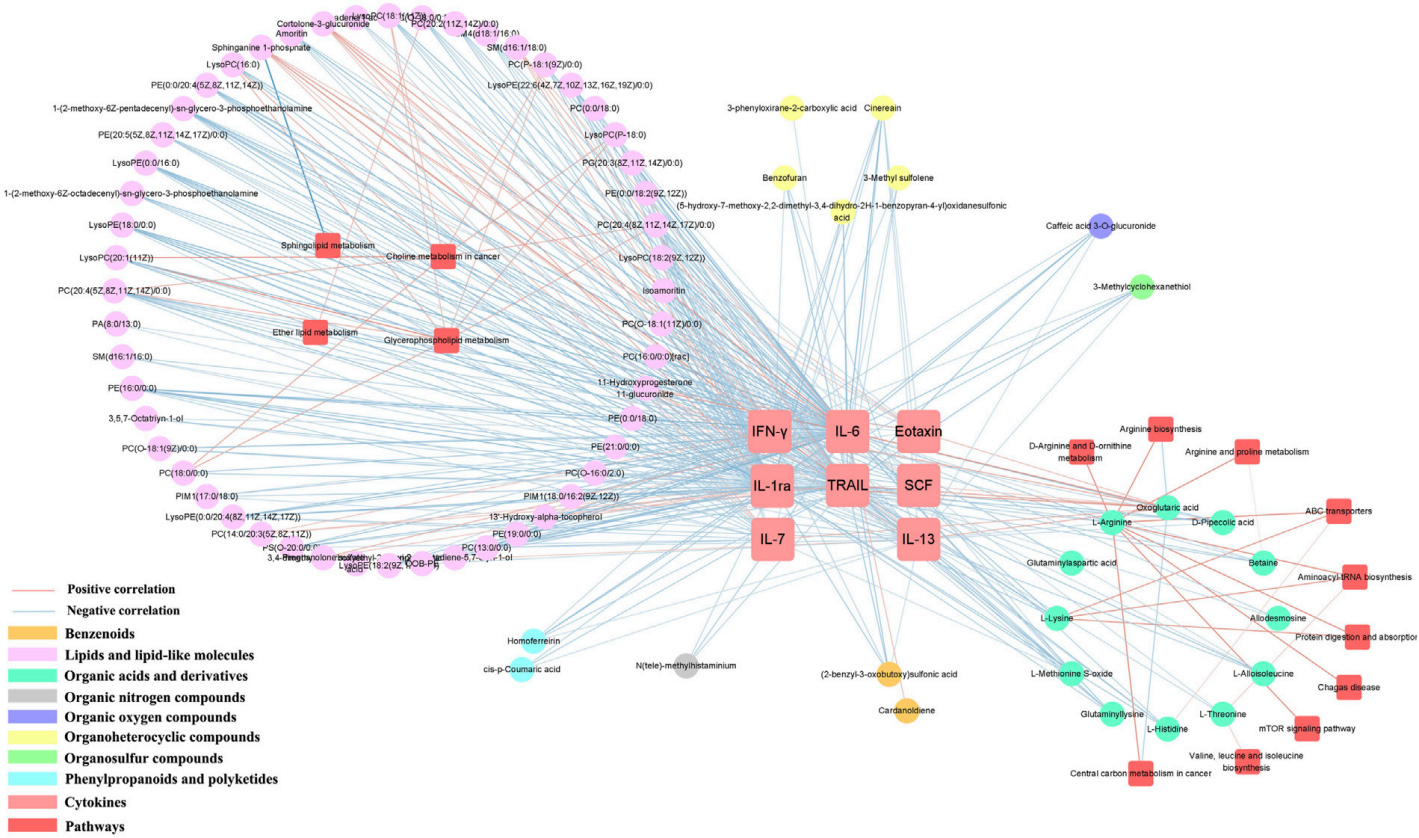
Metabolite-cytokine correlation in serum samples. Only metabolite correlations with |PCC| ≥ 0.9 and *p* < 0.001 were considered.

Most metabolites were also negatively correlated with cytokines, similar to the results of clinical symptom-metabolite correlation analysis. Among the phospholipid metabolites were SM(d16:1/18:0) and sphinganine 1-phosphate; cortolone-3-glucuronide and 11-hydroxyprogesterone 11-glucuronide in steroidal glycoside metabolites; pregnanolone sulfate; fatty acids and conjugates (3,4-dimethyl-5-carboxyethyl-2-furanpentanoic acid); glycerophosphocholines (PC(14:0/20:3 (5Z,8Z, 11Z))); phenylpropanoids and polyketides (3,4,5-trihydroxy-6-{[(6E)-3-oxo-1,7-diphenylhepta-4,6-dien-1-yl]oxy}oxane-2 -carboxylic acid); alkaloids and derivatives (8-carboxymethyldihydrochelerythrine); benzenoids (cardanoldiene); gamma-keto acids and derivatives (oxoglutaric acid and glutaminylaspartic acid) were positively correlated. However, 3,4,5-trihydroxy-6-{[(6E)-3-oxo-1,7-diphenylhepta-4,6-dien-1-yl]oxy}oxane-2 -carboxylic acid were negatively correlated in IL-7 and SCF. (Figure 6).

## 4 Discussion

Influenza virus is a highly contagious respiratory disease that causes high morbidity and mortality worldwide. Although the global COVID-19 pandemic has altered or may reduce the annual circulation of influenza viruses around the world, the influence of influenza viruses cannot be ignored. The influenza syndrome is characterized by fever, headache, cough, sore throat, myalgia, nasal congestion, weakness and loss of appetite. In our study, patients with uncomplicated influenza were enrolled in this trial to evaluate the efficacy of LS. The AUC of the total influenza symptom scores served as the primary outcome to evaluate the efficacy of LS on influenza. The CTCAE was used to assess the adverse effects of LS. The rate of adverse effects in LS group was slightly higher than that in Placebo group, but not statistically different. Our results indicated that the AUC of the total influenza symptom scores in LS group was significantly smaller than that in Placebo group. In addition, the time to relief of flu symptoms in LS group was significantly shorter than that in the Placebo group. Therefore, LS was superior to placebo in alleviating influenza symptoms and benefit influenza with no significant adverse effects. Generally, sore throat is the first symptom and one of major symptom of acute upper respiratory tract viral infections and can develop into more severe sore throat pain related to pharyngitis, nasopharyngitis, or tonsillitis. LS have been used as treatment for pharyngitis, nasopharyngitis or tonsillitis with a long history and benefit patients with pharyngitis (Zhu Guoqin, 2015). Of note, we have confirmed the efficacy of LS in mitigating nasopharyngeal symptoms and nasopharyngeal pathology caused by influenza virus via ferret model (unpublished). The results of this study showed that LS treatment significantly improved symptoms of sore throat, nasal congestion and cough in patients compared with Placebo group. Overall, it showed that LS treatment could effectively relieve respiratory symptoms, shorten the recovery time of symptoms, and improve the prognosis of patients. Therefore, LS could be considered for the treatment of patients with uncomplicated influenza.

We further focused on the changes in non-targeted metabolomics before and after LS intervention. We compared patients in the start- and end- LS groups and showed that there were significant differences in metabolic profiles in serum before and after LS treatment, particularly changes in lipid types, which are consistent with changes in metabolic profiles of LS in healthy human groups (Wang et al., 2022). In both sample groups, the greatest change in the number of lipid metabolites was observed (62.8%), followed by organic acids and derivatives (11.4%) and organ heterocyclic compounds (6.0%).

Lipids are the basic components of cell membranes. Influenza viruses can acquire a host-derived lipid envelope by hijacking host cells for efficient replication and budding. Following influenza virus infection, the levels of phospholipids and several lipid derivatives are disturbed. It has been shown that obese mice induced a more severe inflammatory response to influenza infection than non-obese mice due to abnormal fatty acid and phospholipid metabolism (Milner et al., 2015). A very recent study suggests that phosphatidylcholine (PC) and lysophospholipids (Lyso) are found to be reduced in the blood after influenza virus infection, as well as elevate levels of sphingomyelin (SM) (Tanner et al., 2014; Keshavarz et al., 2020). It suggested a direct link between the influenza-mediated immune response of the organism and the abundance of lipids and their metabolism *in vivo*.

In the study, we identified several types of lipids, mainly including glycerophospholipids, sphingolipids, fatty acyls, steroids and steroid derivatives, sterol lipids. Glycerophospholipids and sphingolipids were important components on biological membranes. Influenza virus could not germinate without viral assembly on the host plasma membrane, and the process of viral assembly was closely related to membrane rafts. A pervious study determined the liposomes of host MDCK cells, including their apical membranes and the influenza virus particles that budded from them (Gerl et al., 2012). This revealed that the apical membrane was enriched in sphingolipids and cholesterol, whereas glycerophospholipids were reduced, and storage lipids were depleted compared to the whole cell membrane. Compared to the donor membrane, the viral membrane further exhibited enrichment in sphingolipids and cholesterol at the expense of phosphatidylcholine (Gerl et al., 2012). In our study, we determined the alternation of 86 glycerophospholipid metabolites between the start- and end- LS groups, of which 82 were upregulated and 4 were downregulated. In addition, 8 sphingolipids were downregulated. This indicated that glycerophospholipids and phospholipid disorders were improved after LS treatment, which may have inhibited the viral assembly process.

Fatty acids are precursors of various bioactive lipids, and different free fatty acid species are involved in the development and progression of influenza. It has been shown that influenza virus infection is associated with reduction in essential fatty acyl and long-chain coenzyme A species metabolites, elevation in cholesterol and arachidonic acid metabolites, and increase expression of the inflammatory cytokines IL-6 and TNF-α. Mitochondrial β-oxidation of long-chain fatty acids in mouse liver was inhibited during infection, leading to impaired fatty acid oxidation and the development of viral hepatitis (Lin et al., 2010; Tarasenko et al., 2015). A very recent study suggests that the antiviral activity of type 1 IFN can be exerted by blocking the synthesis of glucose-derived cholesterol and fatty acids (Blanc et al., 2011). We determined the alternation between the start- and end- LS groups for 11 fatty acyl metabolites, 5 of which were upregulated and 6 downregulated, in addition to all 11 steroid metabolites being downregulated (except kurilensoside H which was upregulated) in our study. The abundance of long-chain coenzyme A compounds (hydroxyisovaleroyl carnitine) increased significantly, while the abundance of arachidonic acid compounds (PGF1a alcohol) decreased significantly in LS-end group. Cytokines such as IL-1ra, IL-6, IL-10, Eotaxin, IFN-γ and TRAIL also decreased in expression in LS-end group. Consistent with the results of our previous experiments, LS inhibited the expression of inflammatory factors such as TNF-α、IL-6、IL-1α、IFN-γ after influenza infection *in vivo* and *in vitro* (Ma et al., 2020).

Intriguingly, influenza virus-induced inflammatory factors play an important role in the development and progression of disease. Influenza virus infection induced the release of inflammatory responses, with IL-6 and TNF-α levels in nasal irrigation fluid peaking early in the infection (day 2), and correlating directly with viral titers, temperature, mucus production, and symptom scores (Hayden et al., 1998). Another study confirmed that inflammatory factors such as CCL2, CCL3, CXCL2 (IL-8), CXCL10, TNF-α and IL-1α in serum were also strongly associated with the development of systemic symptoms such as upper respiratory tract symptoms and fever (Eccles, 2005).

We further correlated the clinical symptoms, inflammatory factors and metabolites of the patients and found that inflammatory factors (IL-1ra, IL-6, IL-10, Eotaxin, IFN-γ, TRAIL), clinical symptoms (fever, headache, sore throat, chills, fatigue) and sphingolipid metabolism, glycerophospholipid metabolism, ether lipid metabolism and amly tRNA synthesis were closely related. Influenza viruses belong to the family Orthomyxoviridae and consist of two layers, the inner and outer. The lipids account for 20–25% of the total weight of the viral particles, making the virus sensitive to ether and other lipid solvents (Gerl et al., 2012). It has been shown that the apical membrane of the host after virus infection was enriched in sphingolipids and cholesterol, while glycerophospholipids and storage lipids were reduced. Whereas sphingolipids were important components of the RNA virus envelope and were involved in the inflammatory response of the organism during viral infection. Among them, SM was one of the sphingolipid metabolites, and exogenous reduction of SM can inhibit influenza virus replication (Audi et al., 2020). ASMase and SPHK1 are the key enzymes of the sphingolipid pathway. On the one hand, they can participate in the inflammatory response promoted by LPS and TNF-α through the SPHK1-NF-κB pathway, inducing the expression of inflammatory factors such as IL-6, TNF-α, IL-1α etc (Xia et al., 2002; Zhi et al., 2006; Hammad et al., 2008). On the other hand, it can also activate the NF-κB cascade response through the ASMase-Cer pathway, which further leads to the expression of inflammation-associated proteins and factors, thus amplifying the inflammatory response (Schutze et al., 1992). Consistent with our finding that SM metabolites were positively correlated with inflammatory factors, while glycerophospholipids were negatively correlated with them. One study showed that Cinobufotalin was one of the active components of LS, and it could inhibit the expression of SPHK1 in A549 cells, thereby suppressing the growth of tumor cells (Cheng et al., 2015). We speculated that LS might improve influenza-like symptoms and excessive inflammatory response after influenza virus infection by regulating phospholipid, glycerophospholipid metabolic pathway.

It is well known that amino acids are used by viruses in host cells to synthesize proteins and replicate, requiring the corresponding transfer RNA (tRNA) and amyl-tRNA synthetase (aaRS). The activity of many aaRS in higher eukaryotes appears to be regulated by the cytoplasmic multi-tRNA synthetase complex (MSC). This large molecular complex consists of eight aminoacyl-tRNA synthetases, including glutamyl-prolinyl-tRNA synthetase (EPRS), leucyl-tRNA synthetase (LRS), lysyl-tRNA synthetase (KRS) and arginyl-tRNA synthetase (RRS), and three auxiliary aaRS-interacting multifunctional proteins, AIMP1 (p43), AIMP2 (p38) and AIMP3 (p18). It has been shown that AIMp1/p43 is able to regulate the aminoacylation activity of tRNA synthetase in normal cells, and it is also involved innate and adaptive antiviral immunity to influenza virus infection in the host (Liang et al., 2018). Upon viral infection, EPRS may protect the mitochondrial antiviral signaling protein (MAVS) by blocking PCBP2-mediated ubiquitination. PCBP2 is a negative regulator of MAVS, and this interaction blocks PCBP2-mediated MAV ubiquitination and ultimately inhibits viral replication (Lee et al., 2016). We speculated that LS might maintain the antiviral immune response by activating MSC and its corresponding ARS-interacting multifunctional proteins.

In summary, our study performed that LS had no significant adverse effects and had an important role in improving influenza-like symptoms and were beneficial for the treatment of influenza. There were significant differences in metabolic profiles in serum before and after LS treatment, particularly changes in lipid types. Viruses need to induce specific host metabolic pathways for replication and propagation. Therefore, LS may play a key role in influenza virus infection by targeting multiple metabolic pathways (phospholipid, fatty acid, arachidonic acid and amyl-tRNA synthesis pathways).

## Data Availability

The original contributions presented in the study are included in the article/Supplementary Material, further inquiries can be directed to the corresponding author.

## References

[B1] Audi A. Soudani N. Dbaibo G. Zaraket H. (2020). Depletion of host and viral sphingomyelin impairs influenza virus infection. Front. Microbiol. 11, 612. 10.3389/fmicb.2020.00612 32425895 PMC7203554

[B2] Blanc M. Hsieh W. Y. Robertson K. A. Watterson S. Shui G. Lacaze P. (2011). Host defense against viral infection involves interferon mediated down-regulation of sterol biosynthesis. PLoS Biol. 9, e1000598. 10.1371/journal.pbio.1000598 21408089 PMC3050939

[B3] Centers for Disease Control and Prevention (2022). Flu symptoms & diagnosis. Avaliable at: https://www.cdc.gov/flu/symptoms/symptoms.htm (Accessed May 19, 2022).

[B4] Cheng L. Chen Y. Z. Peng Y. Yi N. Gu X. S. Jin Y. (2015). Ceramide production mediates cinobufotalin-induced growth inhibition and apoptosis in cultured hepatocellular carcinoma cells. Tumour Biol. 36 (8), 5763–5771. 10.1007/s13277-015-3245-1 25724183

[B5] Dong G. Peng C. Luo J. Wang C. Han L. Wu B. (2015). Adamantane-resistant influenza a viruses in the world (1902-2013): Frequency and distribution of M2 gene mutations. PLoS One 10 (3), e0119115. 10.1371/journal.pone.0119115 25768797 PMC4358984

[B6] Eccles R. (2005). Understanding the symptoms of the common cold and influenza. Lancet. Infect. Dis. 5 (11), 718–725. 10.1016/S1473-3099(05)70270-X 16253889 PMC7185637

[B7] Gerl M. J. Sampaio J. L. Urban S. Kalvodova L. Verbavatz J. M. Binnington B. (2012). Quantitative analysis of the lipidomes of the influenza virus envelope and MDCK cell apical membrane. J. Cell. Biol. 196 (2), 213–221. 10.1083/jcb.201108175 22249292 PMC3265945

[B8] Gowda G. A. Zhang S. Gu H. Asiago V. Shanaiah N. Raftery D. (2008). Metabolomics-based methods for early disease diagnostics. Expert Rev. Mol. diagn. 8, 617–633. 10.1586/14737159.8.5.617 18785810 PMC3890417

[B9] Hammad S. M. Crellin H. G. Wu B. X. Melton J. Anelli V. Obeid L. M. (2008). Dual and distinct roles for sphingosine kinase 1 and sphingosine 1 phosphate in the response to inflammatory stimuli in RAW macrophages. Prostagl. Other Lipid Mediat. 85 (3-4), 107–114. 10.1016/j.prostaglandins.2007.11.002 PMC2290737 18166496

[B10] Hayden F. G. Fritz R. Lobo M. C. Alvord W. Strober W. Straus S. E. (1998). Local and systemic cytokine responses during experimental human influenza A virus infection. Relation to symptom formation and host defense. J. Clin. Invest. 101 (3), 643–649. 10.1172/JCI1355 9449698 PMC508608

[B11] Hu K. Guan W. J. Bi Y. Zhang W. Li L. Zhang B. (2021). Efficacy and safety of lianhuaqingwen capsules, a repurposed Chinese herb, in patients with coronavirus disease 2019: A multicenter, prospective, randomized controlled trial. Phytomedicine. 85, 153242. 10.1016/j.phymed.2020.153242 33867046 PMC7229744

[B12] Keshavarz M. Solaymani-Mohammadi F. Namdari H. Arjeini Y. Mousavi M. J. Rezaei F. (2020). Metabolic host response and therapeutic approaches to influenza infection. Cell. Mol. Biol. Lett. 25, 15. 10.1186/s11658-020-00211-2 32161622 PMC7059726

[B13] Lampejo T. (2020). Influenza and antiviral resistance: An overview. Eur. J. Clin. Microbiol. Infect. Dis. 39 (7), 1201–1208. 10.1007/s10096-020-03840-9 32056049 PMC7223162

[B14] Lee E. Y. Lee H. C. Kim H. K. Jang S. Y. Park S. J. Kim Y. H. (2016). Infection-specific phosphorylation of glutamyl-prolyl tRNA synthetase induces antiviral immunity. Nat. Immunol. 17, 1252–1262. 10.1038/ni.3542 27595231 PMC5173487

[B15] Li Q. Luo N. C. Dai J. J. Di L. Q. Wang H. B. Yang Y. Q. (2019). A method for detecting the Liu Shen pill by HPLC. Chinese patent CN20191012362.8.

[B16] Li T. C. Chan M. C. Lee N. (2015). Clinical implications of antiviral resistance in influenza. Viruses 7 (9), 4929–4944. 10.3390/v7092850 26389935 PMC4584294

[B17] Li Z. J. Zhang H. Y. Ren L. L. Lu Q. B. Ren X. Zhang C. H. (2021). Etiological and epidemiological features of acute respiratory infections in China. Nat. Commun. 12 (1), 5026. 10.1038/s41467-021-25120-6 34408158 PMC8373954

[B18] Liang D. Tian L. You R. Halpert M. M. Konduri V. Baig Y. C. (2018). AIMp1 potentiates TH1 polarization and is critical for effective antitumor and antiviral immunity. Front. Immunol. 8, 1801. 10.3389/fimmu.2017.01801 29379495 PMC5775236

[B19] Lin S. Liu N. Yang Z. Song W. Wang P. Chen H. (2010). GC/MS-based metabolomics reveals fatty acid biosynthesis and cholesterol metabolism in cell lines infected with influenza A virus. Talanta 83, 262–268. 10.1016/j.talanta.2010.09.019 21035673

[B20] Liu Q. Liu J. (2022). Clinical Practice guidelines for the treatment of influenza with Chinese medicine. J. Chin. Med. 63 (01), 85–98.

[B21] Ma Q. Huang W. Zhao J. Yang Z. (2020). Liu Shen Wan inhibits influenza a virus and excessive virus-induced inflammatory response via suppression of TLR4/NF-kappaB signaling pathway *in vitro* and *in vivo* . J. Ethnopharmacol. 252, 112584. 10.1016/j.jep.2020.112584 31972325

[B22] Ma Qinhai C. R. L. B. (2022). Exploring the effect of Liushen capsule on lipopolysaccharide-induced inflammatory response in macrophages RAW264.7 based on NF-κB pathway. J. Chin. Med. 63 (04), 362–369.

[B23] Ma Q. Lei B. Chen R. Liu B. Lu W. Jiang H. (2022). Liushen Capsules, a promising clinical candidate for COVID-19, alleviates SARS-CoV-2-induced pulmonary *in vivo* and inhibits the proliferation of the variant virus strains *in vitro* . Chin. Med. 17 (1), 40. 10.1186/s13020-022-00598-4 35365215 PMC8972667

[B24] Milner J. J. Rebeles J. Dhungana S. Stewart D. A. Sumner S. C. Meyers M. H. (2015). Obesity increases mortality and modulates the lung Metabolome during pandemic H1N1 influenza virus infection in mice. J. Immunol. 194, 4846–4859. 10.4049/jimmunol.1402295 25862817 PMC4417391

[B25] Ministry of Health China (2009). Influenza A (H1N1) treatment plan in 2009. Infect. Dis. Inf. 22 (03), 121–123.

[B26] Nuzzo A. Saha S. Berg E. Jayawickreme C. Tocker J. Brown J. R. (2021). Expanding the drug discovery space with predicted metabolite-target interactions. Commun. Biol. 4, 288. 10.1038/s42003-021-01822-x 33674782 PMC7935942

[B39] Peteranderl C. Herold S. Schmoldt C. (2016). Human influenza virus infections. Semin. Respir. Crit. Care Med. 37 (4), 487–500. 10.1055/s-0036-1584801 27486731 PMC7174870

[B27] Rabinowitz J. D. Purdy J. G. Vastag L. Shenk T. Koyuncu E. (2011). Metabolomics in drug target discovery. Cold Spring Harb. Symp. Quant. Biol. 76, 235–246. 10.1101/sqb.2011.76.010694 22114327 PMC4084595

[B28] Schutze S. Potthoff K. Machleidt T. Berkovic D. Wiegmann K. Kronke M. (1992). TNF activates NF-kappa B by phosphatidylcholine-specific phospholipase C-induced "acidic" sphingomyelin breakdown. Cell. 71 (5), 765–776. 10.1016/0092-8674(92)90553-o 1330325

[B29] Tanner L. B. Chng C. Guan X. L. Lei Z. Rozen S. G. Wenk M. R. (2014). Lipidomics identifies a requirement for peroxisomal function during influenza virus replication. J. Lipid Res. 55, 1357–1365. 10.1194/jlr.M049148 24868094 PMC4076098

[B30] Tarasenko T. N. Singh L. N. Chatterji-Len M. Zerfas P. M. Cusmano-Ozog K. McGuire P. J. (2015). Kupffer cells modulate hepatic fatty acid oxidation during infection with PR8 influenza. Biochim. Biophys. Acta 1852, 2391–2401. 10.1016/j.bbadis.2015.08.021 26319418 PMC6662236

[B40] Veldhuis Kroeze E. Bauer L. Caliendo V. van Riel D. (2021). In vivo models to study the pathogenesis of extra-respiratory complications of influenza A virus infection. Viruses 13 (5), 848. 10.3390/v13050848 34066589 PMC8148586

[B31] Wang C. Cao B. Liu Q. Q. Zou Z. Q. Liang Z. A. Gu L. (2011). Oseltamivir compared with the Chinese traditional therapy maxingshigan-yinqiaosan in the treatment of H1N1 influenza: A randomized trial. Ann. Intern. Med. 155 (4), 217–225. 10.7326/0003-4819-155-4-201108160-00005 21844547

[B32] Wang X. Xu X. Chen Y. Li Z. Zhang M. Zhao C. (2022). Liu shen capsule alters airway microbiota composition and metabolite profiles in healthy humans. Front. Pharmacol. 12, 824180. 10.3389/fphar.2021.824180 35153770 PMC8831732

[B33] World Health Organization (2022). Current influenza update - WHO. Avaliable at: https://www.who.int/teams/global-influenza-programme/surveillance-and-monitoring/influenza-updates/current-influenza-update (Accessed May 19, 2022).

[B34] Xia P. Wang L. Moretti P. A. Albanese N. Chai F. Pitson S. M. (2002). Sphingosine kinase interacts with TRAF2 and dissects tumor necrosis factor-alpha signaling. J. Biol. Chem. 277 (10), 7996–8003. 10.1074/jbc.M111423200 11777919

[B35] Yong Q. (2021). Clinical observation of Liushenwan in the treatment of influenza. Chin. Traditional Herb. Drugs 52 (06), 1687–1691.

[B36] Zhao J. Wang Y. Huang X. Ma Q. Song J. Wu X. (2021). Liu Shen Wan inhibits influenza virus-induced secondary *Staphylococcus aureus* infection *in vivo* and *in vitro* . J. Ethnopharmacol. 277, 114066. 10.1016/j.jep.2021.114066 33766755

[B37] Zhi L. Leung B. P. Melendez A. J. (2006). Sphingosine kinase 1 regulates pro-inflammatory responses triggered by TNFalpha in primary human monocytes. J. Cell. Physiol. 208 (1), 109–115. 10.1002/jcp.20646 16575915

[B38] Zhu Guoqin X. Y. L. S. (2015). Clinical study on the treatment of acute pharyngitis with Liushenwan. China Med. Pharm. 5 (03), 83–85+93.

