# Corrigendum: Investigating the effects of Liushen Capsules (LS) on the metabolome of seasonal influenza: A randomized clinical trial

**DOI:** 10.3389/fphar.2022.1036927

**Published:** 2023-01-04

**Authors:** Qinhai Ma, Ruihan Chen, Jing Zeng, Biao Lei, Feng Ye, Qihua Wu, Zhengtu Li, Yangqing Zhan, Bin Liu, Bojun Chen, Zifeng Yang

**Affiliations:** ^1^ State Key Laboratory of Respiratory Disease, National Clinical Research Center for Respiratory Disease, Guangzhou Institute of Respiratory Health, The First Affiliated Hospital of Guangzhou Medical University, Guangzhou, Guangdong, China; ^2^ Faculty of Innovation Engineering, Macau University of Science and Technology, Taipa, Macao, China; ^3^ The Second Affiliated Hospital of Guangzhou University of Chinese Medicine, Guangzhou, Guangdong, China; ^4^ Guangzhou Laboratory, Guangdong, China; ^5^ State Key Laboratory of Quality Research in Chinese Medicine, Macau University of Science and Technology, Taipa, Macau, China

**Keywords:** influenza virus, liushen capsules, clinical trial, non-targeted metabolomics, Chinese traditional medicines

In the published article, an error was made in the **Title**, “Investigating the effects of Liushen Capsules on the metabolome of seasonal influenza: A randomized clinical trial,” instead of “Investigating the effects of Liushen Capsules (LS) on the metabolome of seasonal influenza: A randomized clinical trial.”

In the published article, there was an error in the **Author** list, and author “Jing Zeng, Qihua Wu and Bojun Chen” were erroneously excluded. The equal contributions of QM, RC, and JZ were also not signposted in the author list. The corrected author list appears below.

“Qinhai Ma^1†^, Ruihan Chen^1,2†^, Jing Zeng^3†^, Biao Lei^1^, Feng Ye^1*^, Qihua Wu^3^, Zhengtu Li^1^, Yangqing Zhan^1^, Bin Liu^1^, Bojun Chen^3*^ and Zifeng Yang^1,4,5*^.”

In the published article, the following affiliation was not included “The Second Affiliated Hospital of Guangzhou University of Chinese Medicine, Guangzhou, China.”

In the published article, there was an error in Table 7 (Adverse events in LS and Placebo group during trial)**.**

**TABLE 7:** Adverse events in LS and Placebo group during trial.

Event	LS (N = 46)	Placebo (N = 44)
Any adverse event	32 (71.11)	26 (59.09)
Vomiting	17 (53.13)	2 (7.69)
Nausea	16 (50.00)	5 (19.23)
Cough and sputum	11 (34.38)	13 (50.00)
Diarrhea	7 (21.88)	7 (26.92)
Stomach discomfort	3 (9.38)	2 (7.69)
Nasal congestion	1 (3.13)	—
Gastrointestinal discomfort	1 (3.13)	1 (3.85)
Pneumonia	1 (3.13)	1 (3.85)
Abdominal rash	1 (3.13)	—
bloating	1 (3.13)	1 (3.85)
Mouth ulcers	1 (3.13)	—
Urine occult blood 3+	1 (3.13)	—
Fatigue	1 (3.13)	1 (3.85)
Subconjunctival hemorrhage	1 (3.13)	—
Upper respiratory tract infection	1 (3.13)	1 (3.85)
Dizziness	1 (3.13)	—
Bacterial Infections	1 (3.13)	—
Sore Throat	1 (3.13)	1 (3.85)
Foreign body sensation in the left eye	1 (3.13)	—
Decrease in white blood cells	—	1 (3.85)
Urinary Infections	—	1 (3.85)
Elevated urine leukocytes	—	1 (3.85)
Stabbing pain in the chest	—	1 (3.85)

The corrected Table 7 and its caption appear below.

A correction has been made to **Author contributions**, Paragraph Number 1503–1509. This statement previously stated:

“QM, ZY conceived and designed the study; QM, RC performed the study and analyzed the data; QM, RC, BLe wrote the paper; ZY, FY, ZL, YZ, BLi supervised the study and revised the manuscript. All authors read and approved the final manuscript.”

The corrected statement appears below:

“QM and ZY conceived and designed the study; BC, QM, RC, JZ, and QW performed the study, collected the clinical samples and analyzed the data; QM, RC, JZ, and BLe wrote the paper; ZY, BC, FY, ZL, YZ, and BLi supervised the study and revised the manuscript. All authors read and approved the final manuscript.”

The authors apologize for this error and state that this does not change the scientific conclusions of the article in any way. The original article has been updated.

