# Supplementary material for: Investigating the effects of Liushen Capsules (LS) on the metabolome of seasonal influenza: A randomized clinical trial
Source: Front Pharmacol. 2022 Aug 11;13:968182. doi: 10.3389/fphar.2022.968182 (PMC9402892; doi:10.3389/fphar.2022.968182)
Supplement: Supplementary file 3 [file DataSheet3.docx]

**Table lists**

**Table 1** Influenza symptom scoring criteria

| **Symptoms** | Scoring Criteria | | | |
| --- | --- | --- | --- | --- |
|  | **0 (Absent)** | **1 (Mild)** | **2 (Moderate)** | **3 (Severe)** |
| **Fever** | ≤37.2℃ | 37.3～37.9℃ | 38.0～38.9℃ | ≥39℃ |
| **Headache** | Absent | Mild pain, occasional onset | Moderate or persistent pain | Severe headache，cannot continue to work |
| **Muscle soreness** | Absent | Mild muscle soreness | Moderate | Severe muscle soreness，cannot continue to work and sleep |
| **Chills** | Absent | A slight sense of aversion to cold, do not need to add clothes | Aversion to cold, need to add clothes | Chills and shivers, need more clothes or  more covers/bedding |
| **Sweating** | Absent | Slight sweating | Moderate | Excessive sweating |
| **Fatigue** | Absent | Lassitude | Grudgingly able to work | Severe fatigue，cannot continue to work |
| **Sore throat** | Absent | Mild sore throat | Pharyngeal dryness, pharyngalgia  and odynophagia | Severe sore throat, dysphagia |
| **Cough** | Absent | Occasionally | Often | Frequent coughing during the day and night，cannot continue to work and sleep |
| **Nasal congestion** | Absent | Nasal incompetence on one side | Bilateral nasal incompetence | Bilateral nasal incompetence， open-mouth breathing, affecting sleep |

**Table 2** General information on participants in the two groups

| Items | LS  (*N*=46) | Placebo  (*N*=44) | *P* value |
| --- | --- | --- | --- |
| Age (year, Mean±Std) | 21.50±3.75 | 21.20±4.42 | 0.7328 |
| Sex (Man | 28 (60.87) | 21 (47.73) | 0.2899 |
| Female) | 18 (39.13) | 23 (52.27) |  |
| Height (cm, Mean±Std) | 168.45±7.50 | 166.33±7.92 | 0.1963 |
| Weight (kg, Mean±Std) | 59.92±11.27 | 55.10±8.75 | 0.0263 |
| BMI (kg/m^2^_,_ Mean±Std) | 21.00±3.08 | 19.81±2.02 | 0.0335 |
| Body temperature (℃, Mean±Std) | 38.48±0.68 | 38.61±0.78 | 0.4322 |
| Current smoker (No.%, Mean±Std) | 6(13.04) | 1(2.27) | 0.1109 |
| Duration of influenza illness (h, Mean±Std) | 22.65±12.18 | 26.55±9.57 | 0.1005 |
| Highest body temperature within 24 hours before enrollment  (℃, Mean±Std) | 38.74±0.52 | 38.92±0.53 | 0.1099 |
| Influenza vaccination history | 46(100.00) | 44(100.00) | - |
| Relevant treatment history | 46(100.00) | 44(100.00) | - |
| Respiratory diseases | 2(4.35) | 0(0.00) | - |
| Other systemic diseases | 1(2.17) | 0(0.00) | - |
| Rapid virus antigen detection | Positive (Flu A) | | 0.3554 |
|  | 45(97.83) | 41(93.18) |  |
|  | Positive (Flu B) | |  |
|  | 1(2.17) | 3(6.82) |  |

**Table 3** The AUC of total influenza symptom scores in two groups of participants (FAS)

|  |  | LS  (N=46) | Placebo  (N=44) | Statistic | *P* value |
| --- | --- | --- | --- | --- | --- |
| AUC | N(Nmiss) | 45(1) | 41(3) | t=4.32 | <0.0001 |
|  | Mean±Std | 245.40±174.08 | 416.78±193.65 |  |  |
|  | 95%CI | (193.10,297.70) | (355.66,477.91) |  |  |
|  | Min~Max | 26.5~862.5 | 72.5~1057.5 |  |  |

**Table 4** change values of sore throat, cough and nasal congestion symptoms from baseline between LS and placebo groups (FAS)

|  | Items | LS  (N=46) | Placebo  (N=44) | Statistic | *P* value |
| --- | --- | --- | --- | --- | --- |
| Sore throat visit 1 | N(Nmiss) | 45(1) | 41(3) | t=2.68 | 0.0090 |
|  | Mean±Std | -0.98±0.87 | -0.44±1.00 |  |  |
|  | 95%CI | (-1.24, -0.72) | (-0.76, -0.12) |  |  |
|  | Min~Max | -3~1 | -3~3 |  |  |
| Sore throat visit 2 | N(Nmiss) | 42(4) | 39(5) | t=0.59 | 0.5557 |
|  | Mean±Std | -1.07±0.92 | -0.95±0.94 |  |  |
|  | 95%CI | (-1.36, -0.78) | (-1.25, -0.64) |  |  |
|  | Min~Max | -3~1 | -3~1 |  |  |
| Cough  visit 1 | N(Nmiss) | 45(1) | 41(3) | t=3.55 | 0.0006 |
|  | Mean±Std | -0.42±0.72 | 0.15±0.76 |  |  |
|  | 95%CI | (-0.64, -0.21) | (-0.09,0.39) |  |  |
|  | Min~Max | -2~1 | -1~2 |  |  |
| Cough  visit 2 | N(Nmiss) | 42(4) | 39(5) | t=3.23 | 0.0018 |
|  | Mean±Std | -0.81±0.80 | -0.23±0.81 |  |  |
|  | 95%CI | (-1.06, -0.56) | (-0.49,0.03) |  |  |
|  | Min~Max | -3~0 | -1~2 |  |  |
| nasal congestion  visit 1 | N(Nmiss) | 45(1) | 41(3) | t=2.27 | 0.0256 |
|  | Mean±Std | -0.47±0.84 | 0.02±1.15 |  |  |
|  | 95%CI | (-0.72, -0.21) | (-0.34,0.39) |  |  |
|  | Min~Max | -2~1 | -2~3 |  |  |
| nasal congestion  visit 2 | N(Nmiss) | 42(4) | 39(5) | t=1.14 | 0.2597 |
|  | Mean±Std | -0.71±0.74 | -0.51±0.85 |  |  |
|  | 95%CI | (-0.95, -0.48) | (-0.79, -0.24) |  |  |
|  | Min~Max | -3~0 | -3~1 |  |  |

**Table 5** The remission time and complete remission time of symptoms in LS and placebo group (FAS)

|  | Items | LS  (N=46) | Placebo  (N=44) | Statistic | P value |
| --- | --- | --- | --- | --- | --- |
| time to alleviation of symptoms | N(Nmiss) | 45(1) | 37(7) | t=2.22 | 0.0296 |
|  | Mean±Std | 30.00±16.98 | 40.51±25.77 |  |  |
|  | 95%CI | (24.90,35.10) | (31.92,49.10) |  |  |
|  | Min~Max | 9~97 | 11~114 |  |  |
| time to complete alleviation of symptoms | N(Nmiss) | 42(4) | 29(15) | t=2.10 | 0.0392 |
|  | Mean±Std | 30.07±17.41 | 39.90±21.91 |  |  |
|  | 95%CI | (24.65,35.50) | (31.56,48.23) |  |  |
|  | Min~Max | 9~97 | 11~87 |  |  |

**Table 6** The rate of number of participants with complete remission of symptoms in LS and placebo group (FAS)

|  |  | LS  (N=46) | Placebo  (N=44) | Statistic | *P* value |
| --- | --- | --- | --- | --- | --- |
| Visit 1 | complete alleviating symptoms | 23(50.00) | 10(22.73) | Exact probability | 0.0090 |
|  | alleviating symptoms, not complete | 23(50.00) | 34(77.27) |  |  |
|  | total | 46(100.00) | 44(100.00) |  |  |
| Visit 2 | complete alleviating symptoms | 42(91.30) | 29(65.91) | Exact probability | 0.0041 |
|  | alleviating symptoms, not complete | 4(8.70) | 15(34.09) |  |  |
|  | total | 46(100.00) | 44(100.00) |  |  |

**Table 7** Adverse events in LS and placebo group during trial

| Event | LS (N=45) | Placebo (N=45) |
| --- | --- | --- |
| Any adverse event | 32(71.11) | 26(59.09) |
| Vomiting | 17(53.13) | 2(7.69) |
| Nausea | 16(50.00) | 5(19.23) |
| Cough and sputum | 11(34.38) | 13(50.00) |
| Diarrhea | 7(21.88) | 7(26.92) |
| Stomach discomfort | 3(9.38) | 2(7.69) |
| Nasal congestion | 1(3.13) | / |
| Gastrointestinal discomfort | 1(3.13) | 1(3.85) |
| Pneumonia | 1(3.13) | 1(3.85) |
| Abdominal rash | 1(3.13) | / |
| bloating | 1(3.13) | 1(3.85) |
| Mouth ulcers | 1(3.13) | / |
| Urine occult blood 3+ | 1(3.13) | / |
| Fatigue | 1(3.13) | 1(3.85) |
| Subconjunctival hemorrhage | 1(3.13) | / |
| Upper respiratory tract infection | 1(3.13) | 1(3.85) |
| Dizziness | 1(3.13) | / |
| Bacterial Infections | 1(3.13) | / |
| Sore Throat | 1(3.13) | 1(3.85) |
| Foreign body sensation in the left eye | 1(3.13) |  |
| Decrease in white blood cells | / | 1(3.85) |
| Urinary Infections | / | 1(3.85) |
| Elevated urine leukocytes | / | 1(3.85) |
| Stabbing pain in the chest | / | 1(3.85) |
