# Supplementary material for: Investigating the effects of Liushen Capsules (LS) on the metabolome of seasonal influenza: A randomized clinical trial
Source: Front Pharmacol. 2022 Aug 11;13:968182. doi: 10.3389/fphar.2022.968182 (PMC9402892; doi:10.3389/fphar.2022.968182)
Supplement: Supplementary file 6 [file DataSheet2.docx]

**Figure Legends**

**Figure 1** Schematic view of participant flow

**Figure 2** Inflammatory factors in the start- and end-LS groups

**Figure 3** Serum metabolomics was used to quantify metabolites in the start- and end- LS groups. **(A)** OPLS-DA plot showing the spatial division between start- and end- LS groups. **(B)** Alignment diagram of correlation coefficients of OPLS-DA model. **(C)** Volcano plot showing the metabolites that differed cumulatively and significantly changed in start- and end- LS groups. **(D)** Heat map of the association between 89 metabolites and start/end LS intervention.

**Figure 4** Pathway analysis was used to enrich the metabolic pathways of differential metabolites start- and end- LS treatment. **(A)** KEGG pathway showing the differential metabolic pathways in start- and end- LS groups. **(B)** Correlation analysis of significantly different metabolites in serum. Only metabolite correlations with |PCC| ≥ 0.9 was considered. Circle indicated metabolites and triangles indicated metabolic pathways, with darker colors indicating higher correlations.

**Figure 5** Metabolite-clinical symptom correlation in serum samples. Only metabolite correlations with |PCC| ≥ 0.9 and *P*<0.001 were considered.

**Figure 6** Metabolite-cytokine correlation in serum samples. Only metabolite correlations with |PCC| ≥ 0.9 and *P*<0.001 were considered.
